# Corrosion Behavior of Steel Reinforcement in Concrete with Recycled Aggregates, Fly Ash and Spent Cracking Catalyst

**DOI:** 10.3390/ma7043176

**Published:** 2014-04-21

**Authors:** Hebé Gurdián, Eva García-Alcocel, Francisco Baeza-Brotons, Pedro Garcés, Emilio Zornoza

**Affiliations:** 1 Civil Engineering Deparment, Universidad de Alicante, Ctra. San Vicente s/n, San Vicente del Raspeig 03690, Spain; E-Mails: h.gurdian@ua.es (H.G.); fbaeza.brotons@ua.es (F.B.-B.); pedro.garces@ua.es (P.G.); 2 Architectural Constructions Department, Universidad de Alicante, Ctra. San Vicente s/n, San Vicente del Raspeig 03690, Spain; E-Mail: eva.garcia@ua.es

**Keywords:** recycled aggregate, spent catalytic cracking catalyst, fly ash, concrete, corrosion, mechanical properties

## Abstract

The main strategy to reduce the environmental impact of the concrete industry is to reuse the waste materials. This research has considered the combination of cement replacement by industrial by-products, and natural coarse aggregate substitution by recycled aggregate. The aim is to evaluate the behavior of concretes with a reduced impact on the environment by replacing a 50% of cement by industrial by-products (15% of spent fluid catalytic cracking catalyst and 35% of fly ash) and a 100% of natural coarse aggregate by recycled aggregate. The concretes prepared according to these considerations have been tested in terms of mechanical strengths and the protection offered against steel reinforcement corrosion under carbonation attack and chloride-contaminated environments. The proposed concrete combinations reduced the mechanical performance of concretes in terms of elastic modulus, compressive strength, and flexural strength. In addition, an increase in open porosity due to the presence of recycled aggregate was observed, which is coherent with the changes observed in mechanical tests. Regarding corrosion tests, no significant differences were observed in the case of the resistance of these types of concretes under a natural chloride attack. In the case of carbonation attack, although all concretes did not stand the highly aggressive conditions, those concretes with cement replacement behaved worse than Portland cement concretes.

## 1. Introduction

Nowadays, the civil engineering industry uses huge quantities of concrete as main structural material. The environmental impact associated to this activity is high due to the extraction of raw materials in quarries, the energy consumption related to cement production and the carbon dioxide emissions released in the fabrication and transportation processes [1–3]. However, society is now sensitive to the problem and this tendency is changing thanks to the efforts of the scientific community and governments.

One of the main strategies to reduce the environmental impact of the concrete industry is the reuse of waste materials. The first approach consisted on replacing variable amounts of cement by industrial by-products with pozzolanic activity. Under this strategy, a number of supplementary cementing materials were tested and developed, such as silica fume, fly ash, ground-granulated blast furnace slag, *etc.* The success of these by-products was absolute, and nowadays it is very usual to use them. Nevertheless, other pozzolans have been successfully implemented in cement products, although their use has not been so extended or many times they have never been applied to real construction. As examples of these minor pozzolans, the following can be listed: rice husk ash [4], sewage sludge ash [5], metakaolin [6], other metal industry slags [7], red sludge ash [8], spent catalytic cracking catalyst [9], calcined clays [10], *etc.* The light usage of these minor pozzolans on the concrete manufacture can be related to the small quantity of them that are produced respect to the total cement demand, the heterogeneity of these materials composition and/or the need of laborious pre-treatments in some cases.

Another way to reduce the contamination derived from the concrete industry is the reuse of recycled aggregate to replace a quantity of the traditional natural aggregate. In this case, environmental considerations can be solidly argued, since neither economical nor technological advantages are easily deduced [11–13], although they exist. In 1978, Nixon [14] made a review about the use of recycled aggregate in concrete. That work scanned the period from 1944 to 1977 and it documented the extended practice of using materials coming from destroyed buildings as aggregate for fresh concrete after the Second World War. Then, the demolition of temporal military facilities also provided a source of concrete able to be used as a coarse aggregate for new concrete. In Nixon’s review it can be found that the first assessments made on crushed concrete revealed that it had a lower density than natural aggregate and the concrete made with recycled concrete showed a lower compressive strength, so it had to be compensated with an excessive amount of cement. Other aspect reflected in that review is the higher water absorption of the recycled aggregate concrete, produced due to the porosity of the cement paste contained in the recycled aggregate. Therefore, main issues affected by the incorporation of recycled aggregate in concrete are well known: the decrease in mechanical properties and workability problems due to the increase in porosity.

The conclusions of another review by Hansen [15] and American Concrete Institute [16] recommended a previous step in the preparation of recycled aggregate concrete consisting on humidifying the recycled aggregate and not using the fraction whose diameter is below 2 mm to compensate the increase in the water absorption. In addition, particular attention has to be paid to the presence of impurities, *i.e*., its proportion and composition. In this way, the main problems arise when recycled aggregates come from demolished buildings due to the presence of bricks and tiles [17]. López-Gayarre *et al.* [18] concluded that compressive strength is affected by the quality of recycled aggregate, and the additives can be used to keep the workability without increasing the w/c ratio and therefore avoiding a reduction in mechanical performance. In this way it has also been observed that recycled aggregate concrete presents higher demand of additives, since an increasing amount of these chemical are absorbed in the recycled aggregate porosity, where they cannot exert any influence on the mix [19,20]. Although a decrease in the mechanical performance of recycled aggregate concrete is usually expected [21], it has been successfully used to produce high strength concrete [22]. In that case the amount of recycled aggregate had to be limited to a 30%. Higher quantities led to proportional decrease in concrete mechanical strength. Similar conclusions were achieved by other authors [23–25], even in the long term [26]. In order to keep compressive strength and workability at the same level than control concretes, Butler *et al.* [27] indicated that the amount of water should be increased between 3.1% and 9.4%, although this type of recipes can be considered rather controversial.

This research has considered the combination of both strategies: cement replacement by industrial by-products and natural coarse aggregate substitution by recycled aggregate. Regarding to the binder, a combination of two pozzolanic materials with complementary properties is proposed in order to increase the level of cement replacement without decreasing the concrete’s performance. Fly ash and spent catalytic cracking catalyst have been selected since the first one has shown low short-term reactivity [28], whereas the latter one offers high initial pozzolanic activity [29]. On the other hand, spent catalyst is a highly water demanding product [30] while fly ash is known as a material which increases workability of fresh concrete [31]. Prior works have combined the use of recycled concrete with pozzolans but not with a mix of pozzolans. For instance, a number of authors used fly ash in recycled concrete and studied its influence in compressive strength, pore size distribution and chloride ingress resistance [25,32–34]. Their conclusions pointed out that mechanical strength decreased as the recycled aggregate content increased. In addition, total porosity and mean pore diameter increased. Chloride ingress resistance was reduced with increasing amounts of recycled concrete aggregate. However, when a 25% of fly ash was used as cement replacement, chloride ingress resistance, mean pore diameter and porosity were improved, although carbonation resistance was reduced [35]. Other works, where no fly ash was used, agreed with these conclusions, obtaining results where an increase in the non-steady-state chloride diffusion coefficient also higher carbonation depth were observed when fine recycled concrete aggregate was used [36]. Regarding the carbonation depth of concretes with recycled aggregates, other authors have obtained different results, observing no influence of the presence of recycled aggregates in the carbonation depth [34]. This scattering on the results may arise from two opposite facts that occur when recycled concrete aggregates are used: on one hand, the higher porosity causes a decrease in the carbonation resistance; on the other hand, a more alkaline matrix may lead to an increase due to the presence of higher quantity of compounds that can react with carbon dioxide and fix it. In addition, the use of fly ash can lead to an increase of the workability, which can be useful to compensate the increased water demand of concrete incorporating recycled aggregates [33], and even self-compacting concrete can be obtained with full replacement of coarse aggregate and partial replacement of fine aggregate by recycled aggregate [37,38].

Regarding to the environmental impact of this work, the total amount of wastes affected by this research is huge. About 160,000 tons per year of spent catalyst are produced, and this quantity is increasing annually at a 5% rate [39]. For fly ash, the numbers are several orders higher: more than 600 million tons are, annually, produced worldwide [40]. In the case of recycled concrete aggregate, these quantities are clearly exceeded: just in the European Union, about 180 million tons per year are produced [41].

The EU has only recently introduced recycling targets for construction and demolition waste. A 70% recycling target was introduced in the new EU Waste Framework Directive 2008/98/EC to be achieved by 2020. It includes only recycling of non-hazardous construction and demolition waste and excludes soil and stone. The total recycling rates vary significantly among the countries, but in general, the rate is quite reasonable (>50%) for 11 countries. Five countries have very high recycling percentages (>70%), already in excess of the 2020 target. Six countries have recycling rates between 50% and 70%, one country between 30% and 50% and six countries below 30%. The most significant policy measures to promote the recycling of construction and demolition wastes have been landfill ban or taxes and source separation mandates [42]. In order to obtain easier achievement of this recycling target it is advisable to increase the value of wastes by finding applications where their use is safe. In this way many research teams are devoted to give the society a frame to apply these wastes without risks.

Therefore, it has to be admitted that a huge effort has been made by the scientific community to study and understand how recycled concrete aggregate can be implemented in structural concrete. Nevertheless, the impact of these actions on the corrosion performance of steel reinforcement has to be assessed. The aim of this research is to evaluate the behavior of concretes with a reduced impact on the environment, with a 50% of cement replacement by industrial by-products and a 100% of natural coarse aggregate replacement by recycled aggregate. The concretes prepared according these considerations have been tested in terms of protection offered against steel reinforcement corrosion under carbonation attack and chloride-contaminated environments. In addition, some mechanical tests at 28 days have been performed in order to show their main characteristics at a standard age.

## 2. Results and Discussion

### 2.1. Mechanical Properties

**Figure 1.**
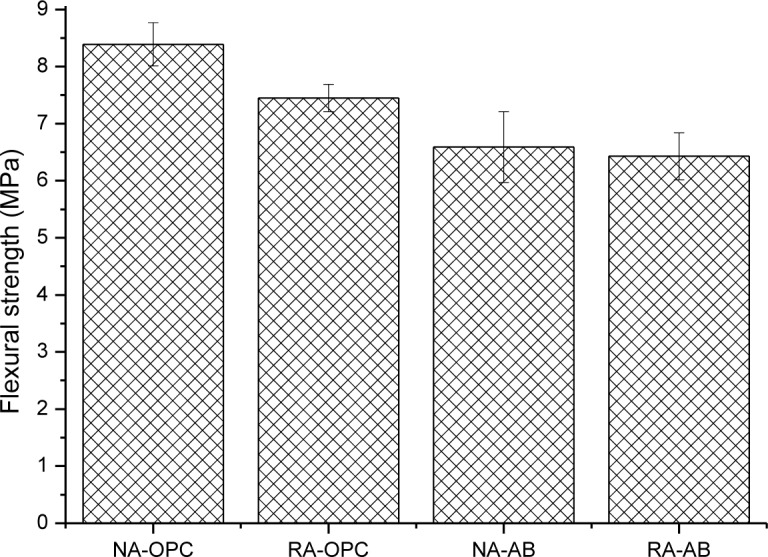
Flexural strength of concrete specimens. Standard deviation is plotted as error bars.

Figure 1 shows flexural strength of concrete specimens at 28 days. It can be observed that total replacement of natural aggregate by recycled one and the use of the alternative binder lead to a reduction in the flexural strength of concrete. Among these factors, the use of the alternative binder causes a more important decrease in this parameter. The use of recycled aggregate does not affect flexural strength if alternative binder has also been used. The values obtained when recycled aggregates have been used (in the range from 6 to 7.5 MPa) are similar to other authors’ results [22] where also total replacement of natural aggregate was applied. Regarding the influence of the alternative binder, other works focused on mortars instead of concrete where this binder combination has been used [43], showed a 20% flexural strength decrease at 28 days due to the presence of these pozzolans, which is similar to the decrease obtained in present research. Although SFCC is a high reactive pozzolan, FA requires longer time to significantly contribute to the strength. In this senses, other works have shown that, at this age, this binder combination has a quantity of alkaline reserve which can be used for FA to recover some strength respect to control concrete in the long term [44]. Therefore, a delayed strength gain may be observed for AB concretes.

**Figure 2.**
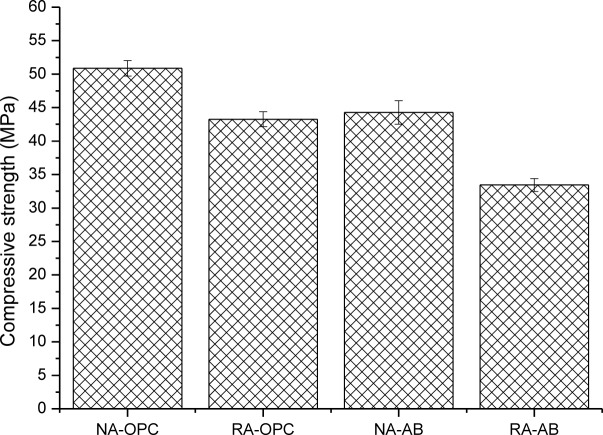
Compressive strength of concrete specimens. Standard deviation is plotted as error bars.

Figure 2 shows compressive strength results of concrete specimens at 28 days of curing time. In this case, again, a decrease in compressive strength is observed for any combination respect to control concrete (NA-OPC). The decrease due to partial cement replacement by FA and SFCC (13.0%) is similar to the reduction produced by aggregate substitution (15.0%). The good behavior shown by AB taking into account the high level of cement replacement may be due to the fact that SFCC is a highly reactive pozzolan that develops the pozzolanic reaction in the short term [29,43–46]. The effect of using RA is almost constant for both binders, since a decrease in 8 and 10 MPa is observed for RA-OPC and RA-AB respect to their respective natural aggregate mixes. This decrease agrees with the general trend observed by other authors, although depending on the particular mix design, the degree of compressive strength loss is different. For example, Corinaldesi [24] registered a 15%–20% of strength loss with just a 30% of natural aggregate replacement by recycled aggregate, whereas Evangelista *et al.* [36] observed a 7% of compressive strength decrease for 100% level of aggregate replacement. In terms of the influence of the pozzolans, previous works offered that this binder may result in compressive strength loss up to 25% [43], so the results collected in this work match the behaviour of the binder.

**Figure 3.**
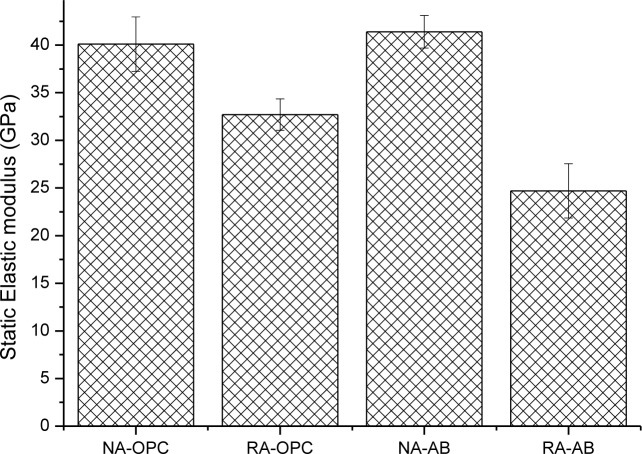
Static Elastic modulus of concrete specimens. Standard deviation is plotted as error bars.

Figure 3 depicts static elastic modulus of concrete specimens at 28 days of curing time. It can be observed that the use of AB does not significantly affect the elastic modulus when natural aggregate is used, whereas a more significant decrease is obtained as a result of the use of RA. This fact could indicate that porosity of concrete incorporating RA is higher with NA concrete, so the density of RA concrete would be lower. The influence of recycled aggregate in static elastic modulus usually shows a negative tendency as in present research. For example, Evangelista *et al.* [36] observed a drop from 35.5 GPa to 28.9 GPa when 100% of aggregate replacement was used, while Chen *et al.* [17] registered a 30% loss. However, other works have obtained no variations in this parameter due to the presence of recycled aggregate [22]. Kou *et al.* [33] observed a decrease similar to that reported in present work and also stated that a 25% of replacement of cement by fly ash did not affect static elastic modulus for natural aggregate concrete. However, and similarly to present results, a decrease in this parameter was observed in recycled aggregate concrete but in a lesser extent, probably due to the higher cement replacement used in actual research.

**Figure 4.**
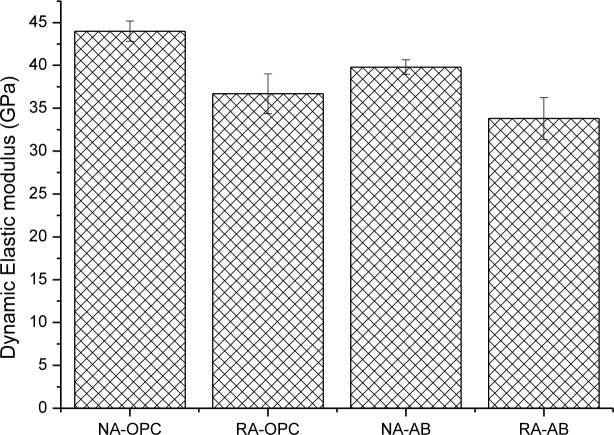
Dynamic Elastic modulus of concrete specimens. Standard deviation is plotted as error bars.

Figure 4 presents dynamic elastic modulus of concrete specimens at 28 of curing time. It can be observed a similar trend to static elastic modulus results, but differences respect to control concrete are lower than in the previous case. Drops of dynamic elastic modulus due to RA presence are 16.6% and 15.1% for OPC and AB concretes, respectively. On the other hand, observed decreases of this parameter due to the use of AB are 9.5% and 7.5% for NA and RA concretes, respectively. Therefore, the type of aggregated exerts more influence on dynamic elastic modulus, and moreover taking into account that AB influence can evolve to higher values in the long term due to FA delayed pozzolanic activity.

**Figure 5.**
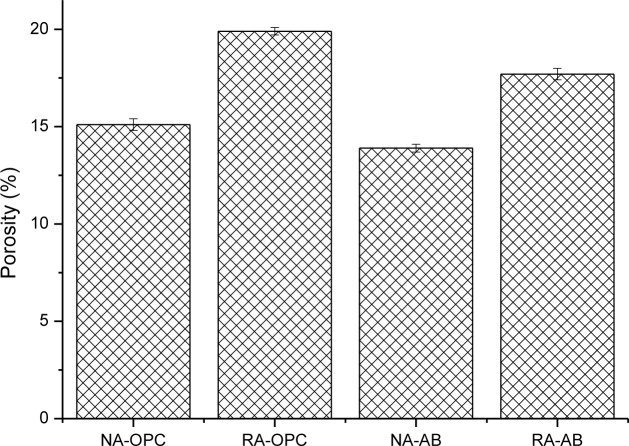
Open porosity of concrete specimens. Standard deviation is plotted as error bars.

Figure 5 shows open porosity values of concrete specimens at 28 days of curing time. Results depicted in Figure 5 are coherent with the hypothesis stated previously relative to elastic modulus of concretes. Open porosity of NA concretes is lower than those of RA concrete. The increase in porosity is due to inherent porosity of recycled aggregate as it has been widely studied by other authors [25,32,33]. In addition, the due to criterion used in this research to apply the coarse aggregate replacement, *i.e*., keeping the coarse aggregate mass constant instead of the volume fraction, in spite of the density difference between RA and NA, produces a higher volumetric proportion of coarse aggregate in RA concrete, resulting in a lower density for them. In this sense, this fact will not only affect mechanical properties of recycled aggregate concrete but also aspects related to durability, as those studied in the second part of this work. In addition it can be observed that the use of the alternative binder slightly reduces the concrete porosity due to the pozzolanic activity of fly ash and spent catalyst.

Summing up, both changes applied to control concrete (aggregate and binder) reduce the mechanical performance of studied concrete. The causes of these facts could be attributed several factors. In the case of the aggregate, lower RA density leads to a decrease of RA concretes density, which can be directly related to their strength losses. Besides, coarse aggregate substitution has been made in mass basis, so taking into account the lower RA density, the binder to coarse aggregate ratio is lower for RA concretes, and then a lower strength is also expected. Therefore, in volumetric terms, in this research, RA concretes have a higher proportion of a less strong component, which joint to high replacement level, leads to a poor mechanical behaviour. In the case of the binder, there is a high substitution degree (50%) and most part of it consists of a pozzolan with slow reaction rate, *i.e*., FA. Thus, at 28 days, the contribution of this part of the binder is not expected to be significant enough, although a positive evolution of strength should be observed in the long term [47], since previous works have shown that there is still a significant amount of portlandite remaining in the binder matrix [44]. Additionally, in order to compensate the lower mechanical performance exhibited by the alternative binder, further studies could consider the efficiency factor of both pozzolans to propose a binder combination without this handicap [47], and taking into account that sufficient amount of portlandite is released by Portland cement to complete the pozzolanic reactions.

### 2.2. Corrosion Performance

**Figure 6.**
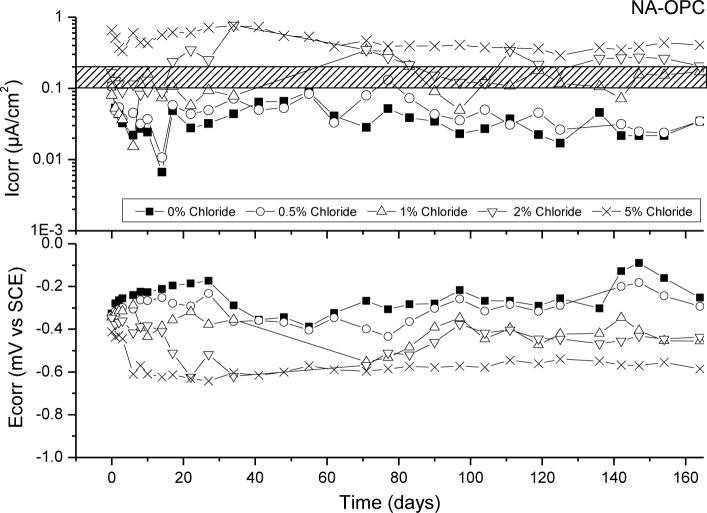
Corrosion rate and corrosion potential of steel reinforcement embedded in NA-OPC specimens and different chloride concentrations.

**Figure 7.**
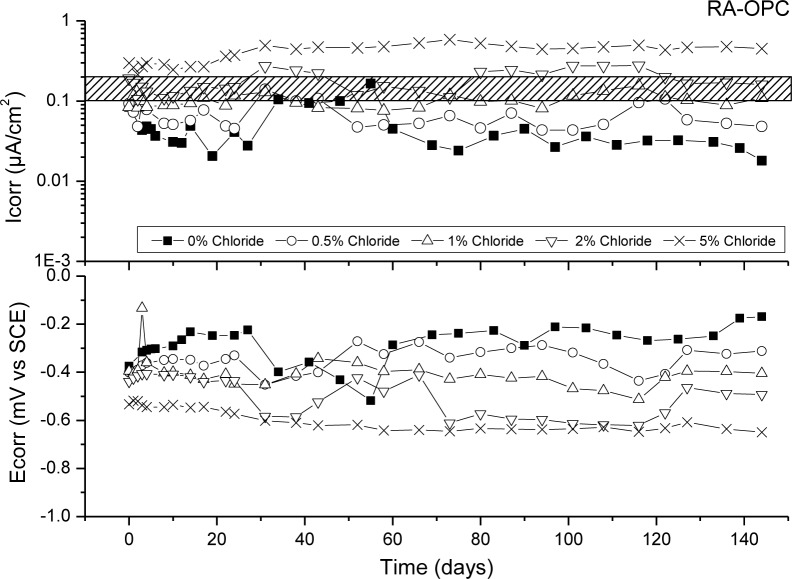
Corrosion rate and corrosion potential of steel reinforcement embedded in RA-OPC specimens and different chloride concentrations.

**Figure 8.**
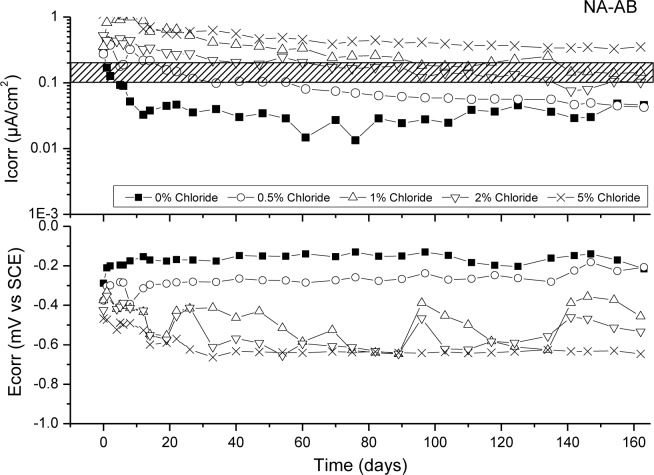
Corrosion rate and corrosion potential of steel reinforcement embedded in NA-AB specimens and different chloride concentrations.

**Figure 9.**
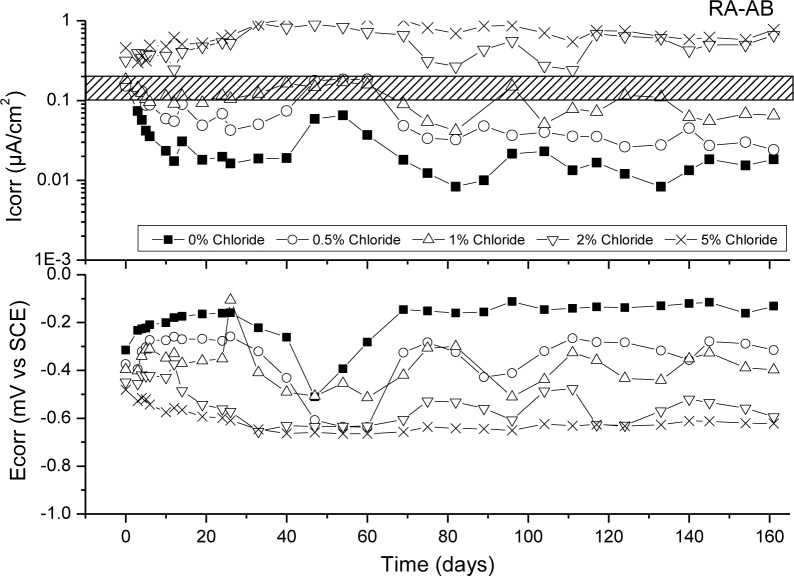
Corrosion rate and corrosion potential of steel reinforcement embedded in RA-AB specimens and different chloride concentrations.

Figures 6–9 presents corrosion rate (*I*_corr_) and corrosion potential (*E*_corr_) evolution of steel reinforcement embedded in different concretes which contain increasing levels of chloride added during the mixing. This chloride addition was tested not to simulated real service conditions but to evaluate the chloride threshold admitted by each matrix (concrete) to stand an acceptable corrosion level. All corrosion graphs present a boundary region between 0.1 and 0.2 μA/cm^2^ that separates the inactive corrosion rate zone from the active corrosion rate zone. Values under 0.1 μA/cm^2^ are considered negligible in terms of durability, while values over 0.2 μA/cm^2^ produce a significant reduction in the service life of the structural element due to the corrosion process [48].

Figure 6 depicts corrosion rate (*I*_corr_) and corrosion potential (*E*_corr_) evolution of steel reinforcement embedded in NA-OPC concrete with different chloride concentrations. It can be observed that chloride threshold for NA-OPC concrete lies between 1% and 2% of chloride, since *I*_corr_ values for 1% lays in the boundary region and 2% slightly exceeds 0.2 μA/cm^2^ almost constantly. *E*_corr_ values are coherent since the higher the *I*_corr_ value, the lower the *E*_corr_ value.

Figure 7 depicts corrosion rate (*I*_corr_) and corrosion potential (*E*_corr_) evolution of steel reinforcement embedded in RA-OPC concrete with different chloride concentrations. In this case, the behaviour exhibited by this concrete is almost the same than NA-OPC concrete, *i.e*., chloride threshold for RA-OPC concrete lies between 1% and 2% of chloride.

Figure 8 shows corrosion rate (*I*_corr_) and corrosion potential (*E*_corr_) evolution of steel reinforcement embedded in NA-AB concrete with different chloride concentrations. The performance of this concrete is better than NA-OPC concrete. In this case the chloride threshold for NA-AB concrete is placed over 2% of chlorides since a clear depassivation of steel is not observed for 2% of chlorides. This behaviour has been previously attributed to higher formation of Friedel’s salt due to a higher Al_2_O_3_ concentration given by FA and SFCC [44,49,50]. Moreover, comparing the *I*_corr_ values registered in NA-AB to control concrete, it can be observed a general reduction for all chloride concentrations.

Figure 9 presents corrosion rate (*I*_corr_) and corrosion potential (*E*_corr_) evolution of steel reinforcement embedded in RA-AB concrete with different chloride concentrations. In this case, the general behaviour is similar to NA-OPC and RA-OPC, establishing a chloride threshold between 1% and 2% of chlorides, although it is clearer now that 2% of chlorides are in the active corrosion zone. It seems that the benefits produced by the use of AB, which traditionally exhibits better resistance to chloride ingress [49] is compensated by the increased porosity of RA concrete which allow higher oxygen diffusion towards steel reinforcement surface and higher corrosion levels. However, it is important to highlight that results of corrosion performance of RA and AB are at least as good as control concrete or even better in NA-AB case. Other authors have obtained an improved performance of steel reinforced concrete with recycled aggregate and supplementary cementing materials respect to control concrete in terms of durability against chloride contaminated environments [51]. However, opposite results have been collected by other authors who point out that initiation period of chloride-induced corrosion is longer for recycled aggregate concrete with fly ash or ground granulated blast furnace slags, but chloride threshold is lower for them [52].

**Figure 10.**
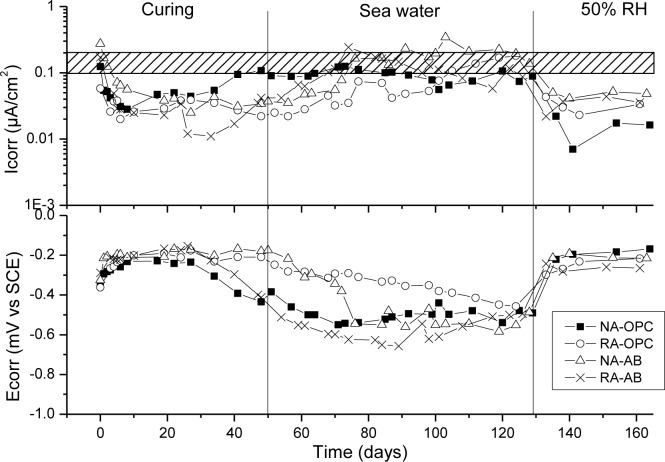
Corrosion rate and corrosion potential of steel reinforcement embedded in different concretes under sea water exposure.

Figure 10 shows corrosion rate (*I*_corr_) and corrosion potential (*E*_corr_) evolution of steel reinforcement embedded in all concrete types when they are exposed to an external chloride attack. This experiment is divided in three stages. The first one is the curing period, to evaluate if steel reinforcement has achieved a passive state. It can be observed that all specimens were correctly passivated before they were subjected to sea water attack. During the partial immersion in sea water solution (second stage), steels of all specimens experienced an increase in *I*_corr_ values and a consequent decrease in *E*_corr_. However a clear disruption of the passive state was not observed for any specimen, so all of them stood the aggressive condition for the 80 days that they were in the sea water solution. The results indicate that the chloride concentration provided by the sea water solution was not high enough to produce a change in the steel surface in that period of time. The changes observed in *I*_corr_ and *E*_corr_ reflected that certain amount of chloride reached the steel surface. A reduction of the partial pressure of oxygen on the steel surface, as a consequence of the immersion, is also deduced. The third stage aims to evaluate the damage on the steel passive film. The increase in the oxygen concentration available to develop the corrosion process while the depassivating agent has actuated usually shows if a significant damage on the steel conditions has been done. In this case, it can be observed that all specimens returned in low corrosion rate values and initial corrosion potentials, so it can be concluded that all concretes protected steel reinforcements under this type of attack.

**Figure 11.**
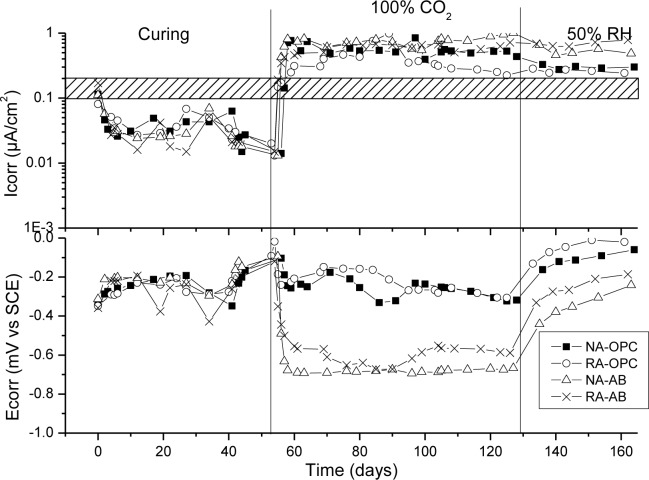
Corrosion rate and corrosion potential under accelerated carbonation exposure.

Figure 11 shows corrosion (*I*_corr_) and corrosion potential (*E*_corr_) evolution of steel reinforcement embedded in all concrete types when they are exposed to a carbonation attack. This experiment is also divided in three stages similar to sea water attack. The first one is the curing period, to evaluate if steel reinforcement has achieved a passive state, and again, this starting condition was correctly attained. Second stage consisted in exposing the steel reinforced specimens to a 100% CO_2_ atmosphere with a 65% RH. In this environment, all specimens showed an abrupt increase in *I*_corr_ at the same time that a decrease in *E*_corr_ values are registered. This indicated that carbonation front reached steel surface and general corrosion of steels started. Among different concretes, no significant differences in the time lapse between the initiation of the attack and the increase of the corrosion rate were observed. Therefore, similar carbonation resistance of all concretes could be deduced, although the high CO_2_ concentration used does not allow to effectively discriminate this property. In the last stage, almost no changes were monitored in *I*_corr_ and *E*_corr_ values, so an effective depassivation was achieve for all specimens. In addition, it can be observed that AB specimens registered higher *I*_corr_ values, indicating worse general performance against this type of aggressive environment. This result is somehow expected since AB’s alkaline reserve, in case of existing, should be significantly lower than OPC concretes’ [43], due to the pozzolanic reaction, so a higher carbonation rate and a lower carbonation-induced densification of the matrix should be observed. On the other hand there is a general consensus about the lower carbonation resistance offered by recycled aggregate concrete [53] which is attributed to the higher permeability of the recycled aggregates on account of the presence of old mortar adhering to the original aggregate, and the old interfacial transition zone (ITZ) between them [54]. Focusing on the corrosion response to the carbonation phenomenon, our results agree with other studies where also supplementary cementing materials were used, finding an increase in the final corrosion density in recycled aggregate concrete with silica fume and fly ash [55].

## 3. Experimental Program and Materials

### 3.1. Materials and Sample Fabrication

**Figure 12.**
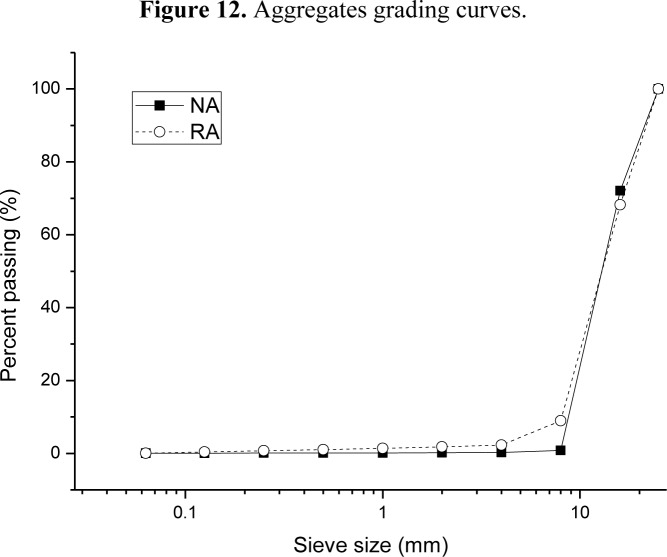
Aggregates grading curves.

Portland cement type used was CEM I 52.5 R, designated according to the European Standards [56]. Fly ash, supplied by Andorra thermoelectric power plant (Teruel, Spain), and spent cracking catalyst, supplied by BP Oil España (Castellon, Spain), were used as replacements of part of the cement. Two types of binders have been used to prepare concrete specimens: ordinary Portland cement (OPC) and an alternative binder (AB) composed by 50% of ordinary Portland cement, 35% of fly ash (FA) and 15% of spent cracking catalyst (SFCC). The chemical composition of cement and pozzolans used are shown in Table 1. SFCC was mechanically activated by grinding during 15 min. A superplasticizer, based on melamine formaldehyde, commercially known as Sikament FF, was used as an admixture in 1.3% to 1.6% proportion of binder mass, depending on the mix demand to keep a constant workability. Workability has been determined through slump value, which was obtained by the Abrams cone method [57]. Two types of coarse aggregates were used to prepare different concrete types: crushed natural limestone (NA) and recycled aggregate obtained from mass concrete demolition wastes (RA), supplied by Holcim Morteros, S.A. (Alicante, Spain). The main properties of natural and recycled aggregates can be found in Table 2. Figure 12 shows grading curves of both aggregates. Recycled concrete used in this work comes from mass concrete, and the supplier certifies the following impurities: Clays < 5%, light particles < 1%, asphalt < 1%, other impurities < 1%.

Four types of concrete were prepared in order to evaluate the effect of the recycled aggregate and supplementary cementing materials, according to the mix proportions shown in Table 3: NA-OPC, used as reference concrete with natural aggregate and Portland cement; RA-OPC, with a total replacement of natural aggregate by recycled aggregate; NA-AB, with natural aggregate and the alternative binder whose composition has been pointed out previously; and, RA-AB with total replacement of natural aggregate by recycled aggregate and the alternative binder. A constant effective water to binder ratio of 0.45 was used. Effective water results from subtracting water absorbed by coarse aggregates from total water. In this way, the increased water absorption produced by recycled aggregates is corrected. Total coarse aggregate replacement has been considered in mass basis, so according to aggregate densities presented in Table 2, a different volumetric fraction of coarse aggregate is obtained in the concrete mix.

The mixing was prepared on a rotatory concrete mixer and the procedure was the following: firstly, the coarse aggregate was placed in the mixer with the plasticizer with one third of total water (according to the technical sheet of the additive); secondly, fine aggregate and binder, and finally the resting amount of water. The fresh concrete was mixed for 10 min.

Six prismatic and six cylindrical specimens of each concrete type were casted. Three prismatic specimens of 100 mm × 100 mm × 400 mm dimensions were used for determining flexural strength and the rest of them for measuring dynamic elastic modulus. Three cylindrical specimens of Ø150 × 300 mm dimensions were used for compressive strength and the rest of them for determining static elastic modulus tests. Both prismatic and cylindrical specimens were cured in moist room (95% RH, 23 °C) until tested [58].

**Figure 13.**
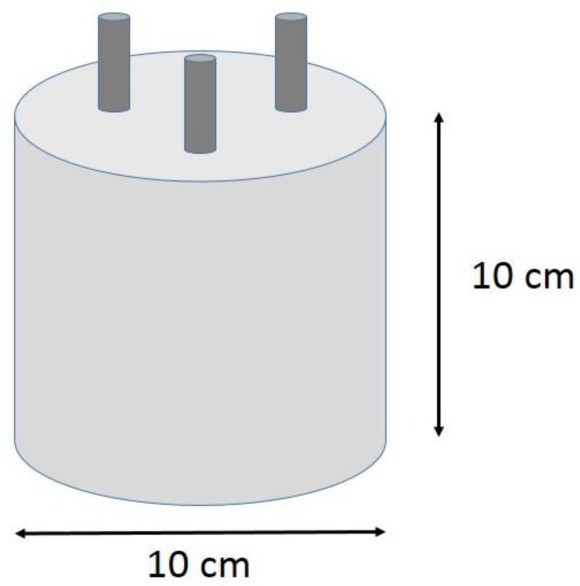
Concrete specimen scheme for corrosion tests. Dimensions in cm. Concrete cover = 3 cm. Distance between electrodes = 3 cm.

In addition, corrosion specimens similar to those reported in previous works [59,60] were prepared. Corrosion specimens were cylinders of 100 mm diameter and 100 mm height (Figure 13). Three embedded corrugated steel bars (as used in construction industry) of 10 mm nominal diameter were the working electrodes and a stainless steel mesh was placed in the contour of the specimen to be used as counter-electrode. The active corrosion area in each of the working electrodes was 28.26 cm^2^. After the 50 days curing period in humid chamber (95% RH, 23 °C), corrosion test specimens were partially immersed in NaCl 0.5 M solution (simulating seawater) or carbonated in a chamber with 100% CO_2_ and 65% RH. The curing period was long enough to ensure a steady passive state prior to aggressive conditions attack. Alternatively, different corrosion specimens were prepared adding different amounts of chlorides during the mixing, ranging from 0% to 5% respect to binder mass.

### 3.2. Mechanical Tests

At 28 days, three cylindrical specimens of each concrete were tested to determine the compressive strength according to the standard test method ASTM C 39/C 39 M [61].

In order to determine the elastic properties of the cylindrical concrete specimens, under longitudinal compressive stress, the Ohmic Extensometry technique was used on the rest of cylindrical specimens. An HBM Spider 8–600 Hz equipment was used jointly with HBM strain gauges (120 Ω, *k* = 2.10) and Catman v.5.0 analysis software. A press machine of 3500 kN capacity was used for both compressive strength and elastic properties tests. Longitudinal and transverse strain values were obtained for each loading cycle up to a maximum value when the applied load is equal to 40% of the sample ultimate load, as specified by the standard test method ASTM C 469 [62]. Tests were performed for every concrete type at 28 days. The static modulus of elasticity was calculated.

At 28 days, three prismatic specimens of each concrete were tested to determine the flexural strength according to the standard test method ASTM C 78 [63]. A hydraulic press machine of 1500 kN capacity was used for these tests.

Resonant frequencies were measured following the standard test method ASTM C215-08 on the rest of prismatic specimens with dimensions 100 mm × 100 mm × 400 mm for the purpose of calculating dynamic Young’s modulus of elasticity. An Erudite MK3 (CNS Farnell) test device of working frequency range 1 Hz to 100 kHz, with EMAT vibrator and piezoelectric receiver was used to perform the test. The resonant frequencies were determined following the forced resonance method, in which, the specimen is placed in a special device with three support points on its base and it is forced to vibrate by an electro-mechanical driving unit. At least 10 readings of the resonance frequency were made in each sample. The dynamic Young’s modulus of elasticity is calculated from the fundamental longitudinal frequency, mass, and dimensions of the specimen tested. The values obtained by this method are, in general, greater than the static modulus of elasticity obtained on cylindrical specimens.

### 3.3. Porosity Tests

Three samples of each type of concrete were dried at 50 °C in a furnace until constant mass was reached, and dry weight (*W*_dry_) was determined. Then samples were water saturated according to ASTM 1202-97 [64]. After completing the saturation process, samples were weighted in a hydrostatic scale to determine submerged weight (*W*_sub_). Finally, the surface of the samples was dried and saturated weight (*W*_sat_) was determined. Open porosity was calculated by the Equation (1).

$$Open\;Porosity\;(\%)=\frac{W_{\text{sat}}-W_{\text{dry}}}{W_{\text{sat}}-W_{\text{sub}}}\times 100$$ (1)

### 3.4. Corrosion Tests

The electrochemical technique used to assess the corrosion rate was the Polarization Resistance (*R*_p_) [48,65,66]. The *R*_p_ is the slope of the polarization curve around the corrosion potential (*E*_corr_): *R*_p_ = Δ*E*/Δ*I* when Δ*E*→0. The *R*_p_ value is related to *I*_corr_ by means of a constant, denominated B by Stern [65] (Equation (2)).

$$I_{\text{corr}}=\frac{B}{R_{p}}$$ (2)

The constant *B* is related to Tafel’s constants β_a_ and β_c_ by the Equation (3).

$$B=\frac{\beta_{a}\cdot \beta_{c}}{2.303\cdot (\beta_{a}+\beta_{c})}$$ (3)

It was stated by Stern [67] that when using a mean *B* value of 26 mV, the maximum *I*_corr_ error factor is 2. For the case of steel embedded in concrete, a value of 26 mV was found [68] for the active state (corrosion) whereas *B* = 52 mV is more appropriate for passive steel. The electrode polarization has been made from *E*_corr_ − 10 mV to *E*_corr_ + 10 mV at a scan rate of 0.5 mV/s. *R*_p_ has been calculated as the slope of the lineal fitting of the polarization curve.

*R*_p_ and corrosion potential were periodically measured during the time of the experiment. Corrosion rate measurements were made by a scanning potentiostat model EG&G 362 from Princeton Applied Research. The reported results are the average of three identical bars. Although it may be controversial to average *E*_corr_ values in the presence of passive/pitting/active transitions no significant scatter between replicate electrodes was found. In order to validate the electrochemical *I*_corr_ values obtained through *R*_p_ measurements, they were compared by means of Faraday’s law to the gravimetric losses obtained in the same rebars.

## 4. Conclusions

The following conclusions can be drawn from this research:

- The use of total natural coarse aggregate replacement by recycled aggregate and 50% of OPC substitution by 15% of SFCC and 35% of FA reduced the mechanical performance at early ages of concretes in terms of elastic modulus, compressive strength, and flexural strength;
- The loss of properties may be attributed more specifically to recycled aggregate than the use of alternative binder;
- An increase in open porosity was observed which is coherent with the changes observed in mechanical tests when recycled aggregate was incorporated;
- Regarding the influence on the performance against most usual corrosion processes, no significant differences were observed in the case of the resistance of these types of concretes under a natural chloride attack;
- In the case of carbonation attack, although all concretes did not stand the highly aggressive conditions, those concretes with cement replacement showed higher corrosion values than OPC concretes.

## Further Investigations

In order to improve the understanding of these materials in terms of its durability, a deeper study of the microstructure evolution should be addressed. These studies may include long term mechanical performance, evolution of porosity, permeability and pozzolanic reaction. In addition, determination of carbonation and chloride ingress kinetics would be valuable to perfectly characterize corrosion protection offered by the proposed materials.

## Figures and Tables

**Table 1.** Chemical composition of binders used in this research (% mass).

%	CaO	SiO_2_	Al_2_O_3_	MgO	Fe_2_O_3_	SO_3_	Na_2_O	K_2_O	LOI	N.D	Specific weight (kg/m^3^)
OPC	62.87	20.21	4.94	1.05	2.85	3.37	0.10	0.95	2.34	1.32	3100
SFCC	0.11	46.04	47.47	0.17	0.58	0.02	0.30	0.02	1.50	3.79	2700
FA	9.83	40.94	24.65	1.59	13.59	1.60	0.34	1.40	2.44	3.62	2800

LOI = Lost on ignition; N/D = Not determined.

**Table 2.** Main properties of natural and recycled aggregate.

Property	Standard used	NA	RA
Classification	UNE-EN 12620	AG-12/20-T-C	AG-12/20-T-R
Apparent density (kg/m^3^)	UNE-EN 1097-6	2710	2490
Water absorption (%)	UNE-EN 1097-6	0.5	4.6
Nominal size (mm)	UNE-EN 933-1	20	20
Los Angeles coeff. (%)	UNE-EN 1097-2	21.1	30.5

**Table 3.** Mix proportions for the concretes evaluated.

Notation	Constituents (kg/m^3^)							Plast. (% binder)	Slump (mm)
	Cement	FA	SFCC	Water	Fine aggregate	NA	RA		
NA-OPC	380	0	0	181	934	865	0	1.3	93
RA-OPC	380	0	0	215	934	0	865	1.6	91
NA-AB	190	133	57	181	934	865	0	1.5	87
RA-AB	190	133	57	215	934	0	865	1.5	88
